# Cell- and subcellular organelle-targeting nanoparticle-mediated breast cancer therapy

**DOI:** 10.3389/fphar.2023.1180794

**Published:** 2023-04-06

**Authors:** Xue Wei, Ming Yang

**Affiliations:** ^1^ Department of Breast Surgery, General Surgery Center, The First Hospital of Jilin University, Changchun, China; ^2^ Key Laboratory of Polymer Ecomaterials, Changchun Institute of Applied Chemistry, Chinese Academy of Sciences, Changchun, China

**Keywords:** organelle-targeting nanoparticle, controlled drug delivery, reversal of drug resistance, inhibition of metastasis, breast cancer therapy

## Abstract

Breast cancer (BC) is the most prevalent malignant tumor, surpassing lung cancer as the most frequent malignancy in women. Drug resistance, metastasis, and immune escape are the major factors affecting patient survival and represent a huge challenge in BC treatment in clinic. The cell- and subcellular organelle-targeting nanoparticles-mediated targeted BC therapy may be an effective modality for immune evasion, metastasis, and drug resistance. Nanocarriers, efficiently delivering small molecules and macromolecules, are used to target subcellular apparatuses with excellent targeting, controlled delivery, and fewer side effects. This study summarizes and critically analyzes the latest organic nanoparticle-mediated subcellular targeted therapeutic based on chemotherapy, gene therapy, immunotherapy, and combination therapy in detail, and discusses the challenges and opportunities of nanoparticle therapy.

## Graphical Abstract

**Figure:**
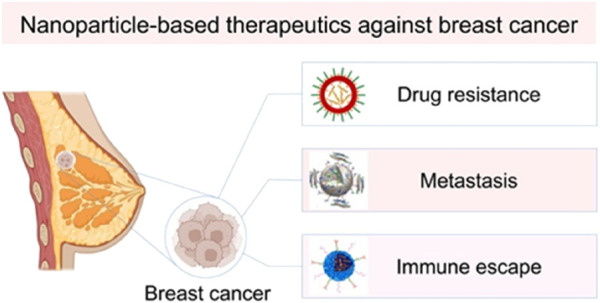
This review summarizes and critically analyzes the latest therapeutic research on nanoparticle-mediated delivery systems targeting breast cancer drug resistance, metastasis, and immune escape in breast cancer and discusses the challenges and opportunities of nanoparticle-based therapy.

## 1 Introduction

Breast cancer (BC) is the most prevalent of malignant tumor in women worldwide (Sung et al., 2021). Treatment modalities for BC, including surgery, radiation therapy, and drug therapy have contributed to an increase in 5-year survival rates for patients. However, these approaches are also difficult to reduce the incidence of metastasis. Advances in cancer research and systems biology have revealed that cancer features often intersect and act synergistically. Moreover, some cancer cells are drug resistant during metastasis. Drug resistance and metastasis are two major obstacles to achieving good treatment outcomes in BC.

Drug resistance during BC treatment often occurs during chemotherapeutic agents or endocrine therapy. Although chemotherapy and hormone therapy are excellent techniques for improving survival rates, they have significant disadvantages. Increased doses of drugs or combinations of drugs required to effectively control cancers, especially that in advanced stages, exacerbate toxicity. Acute and long-term side effects adversely impact the patients’ quality of life (Cai et al., 2010; Dao and Hanson, 2012). Moreover, prolonged use of endocrine drugs can predispose toward drug resistance. Acquired resistance to endocrine therapy has received increasing attention in recent years, with intrinsic mechanisms including somatic alterations, epigenetic alterations, and alterations in the tumor microenvironment (TME) (Hanker et al., 2020). Considering the shortcomings of traditional treatment methods, actively finding effective new methods is necessary.

Metastasis breast cancer (MBC) is incurable and has a high BC mortality rate (Harbeck et al., 2019). Although metastasis usually occurs several years after the primary tumor is diagnosed about 30%, 6% of new BC cases are initially metastatic (O'Shaughnessy, 2005). MBCs are difficult to detect and treat due to their small size, heterogeneity, and dispersion. Distant metastases of BC spread to distant organs through the blood and lymphatic system, thereby increasing the difficulty of treatment. BC often metastasizes to different organ sites, including the bone, lung, liver, brain, and lymph node. Overcoming tumor recurrence, metastasis and drug resistance is the goal of both local and systemic therapies.

Nanoparticles have emerged as promising drug carriers in BC treatment. Nanoparticles offer advantages, such as improved biocompatibility, multifunctional encapsulation of active substances, prolonged blood circulation, active or passive targeting, surface modification, and lower side effects (Adair et al., 2010). Multifunctional smart nanoparticles could be created by manipulating molecules at the nanoscale to address drug deficiency and treat primary breast cancer (PBC) and MBC. Most chemotherapy drugs are hydrophobic, and thus, unsuitable for intravenous delivery. The use of nanoparticles in cancer therapy lengthens the half-life and solubility of drugs, enhancing drug bioavailability (Peer et al., 2007; Wang et al., 2008; Hanafi-Bojd et al., 2015). Additionally, nanoparticles increase the permeability and retention of medication within cancer tissues (Fang et al., 2011). The delivery of nanomaterials can decrease the dose by achieving pharmacologically efficacious concentrations at lower concentrations. Moreover, nanomedicines can be combined with other drugs to reduce side effects and exert synergetic therapeutic effects (Zhang et al., 2022). These advantages have made nanomedicines a hot spot for BC treatment.

Nanomaterials also have great advantages in gene therapy. Genes involved in uncontrolled growth, metastasis, and resistance to drug therapy are often mutated, amplified, or overexpressed in BC. Monoclonal antibodies or small molecule inhibitors are used in targeted therapy to block the vital functions of oncoproteins and reverse the cancer phenotype. However, monoclonal antibodies are difficult to be taken up by cells, and the therapeutic effect of small molecule inhibitors is often unsatisfactory. The advantages of small interfering RNAs (siRNAs) are more obvious, but the main obstacle is overcoming the problem of delivery of siRNA molecules to the cytosol of tumor cells. Nanoparticle as siRNA carriers provide advantages such as increased cellular uptake and integration into components with specific functions (Ngamcherdtrakul and Yantasee, 2019). The development of siRNA therapeutics is mostly in the area of undruggable targets and has advantages in the area of drug targets. Nanomaterials with siRNA cocktails targeting several pathways can be delivered with less restrictions and better therapeutic efficacy simultaneously. Nanomaterials overcome the disadvantages of monoclonal antibodies, small molecule inhibitors, siRNAs, and can be used in combination, thereby opening the door to new therapeutic possibilities.

Nanomaterials crosses the biological barrier to achieve targeted and precise therapy. The intelligent and multifunctional nanoparticles can cross biological barriers and release inside or outside the target cell; finally, reaching the target. Nanoparticles are frequently used to establish and visualize cancer cells as well as deliver therapeutic medicines to prolong survival. This is done by using their unique chemical abilities. Targeting molecules, medicinal substances, fluorophores, or radioisotopes are coupled with nanoparticles in a single formulation. For *in vivo* navigation to cancer cells, biological targets, such as human epidermal growth factor receptor 2 (HER2), can be chemically attached to nanoparticles.

The results achieved by cancer nanomedicine in the past decades are encouraging. Seven nanomedicines have been approved and more than 20 have entered preclinical evaluation for BC (Jiang et al., 2022). Due to the high drug load, extended blood circulation period, decreased enzyme-mediated drug degradation, and sustained drug release, nanocarrier systems function better than drug–ligand conjugation (Li et al., 2014). However, the development process encounters bottlenecks, such as the relative lack of clinical agents for BC relapse, resistance, and metastasis, are mostly stuck in preclinical studies.

**SCHEME 1:**
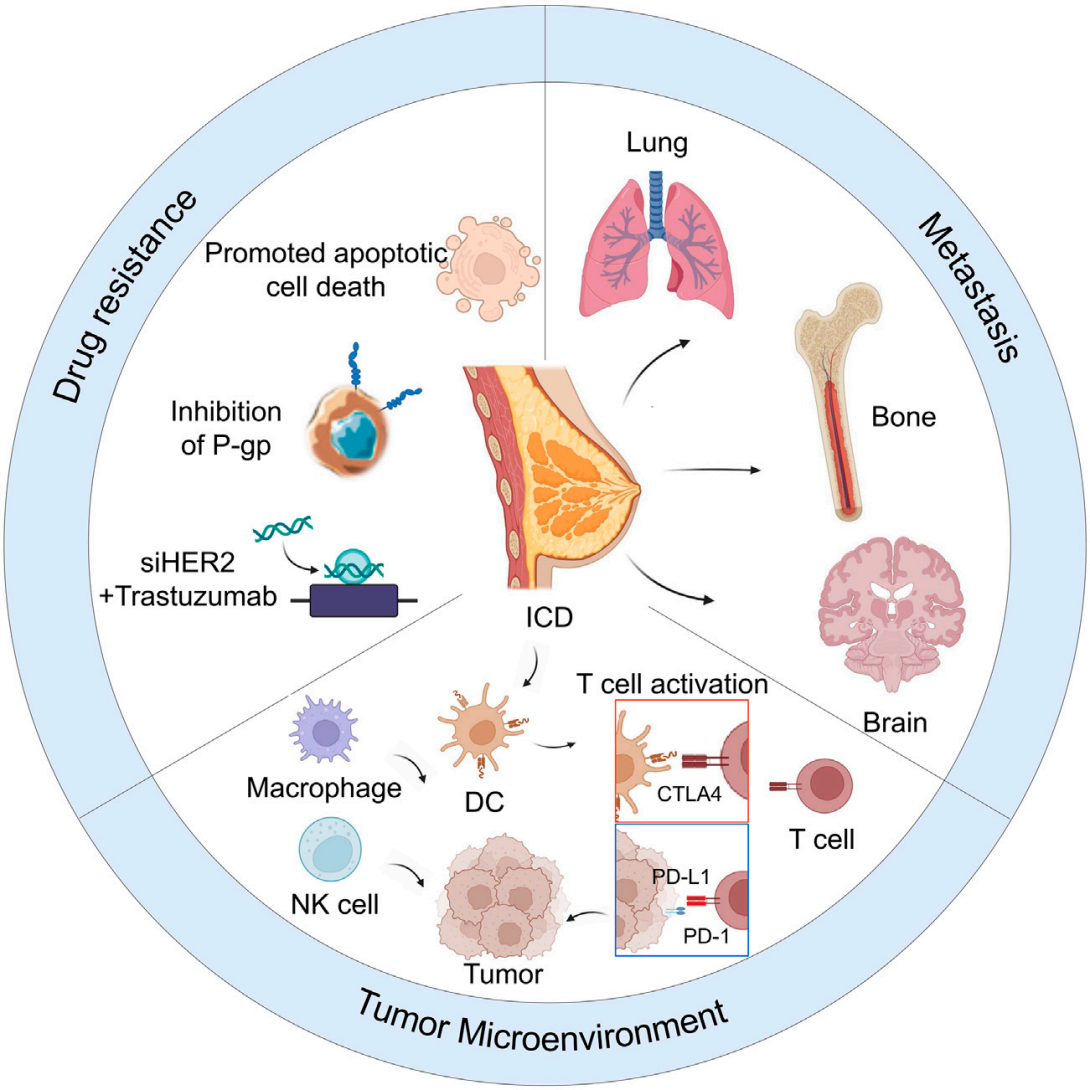
Nanoparticle-based therapy for reducing drug resistance, metastasis, and remodeling of tumor microenvironment in breast cancer.

This review discusses the challenges of treating drug resistance and MBC and presents advances in nanoparticle-based therapy (Scheme 1). Targeted therapy of PBC and MBC, nanomaterials based on chemotherapy drugs, and siRNA and antibody delivery are introduced. Additionally, the nanoparticle-mediated modulation of TME induced to trigger anticancer immune responses for managing PBC and MBC is examined. Finally, insights are offered on the production and use of nanoparticles to accelerate the development of nanoparticle therapy for BC.

## 2 Nanoparticles overcome the challenges of breast cancer chemotherapy

### 2.1 Nanoparticles overcome chemotherapeutic resistance

Owing to the heterogeneity and dispersed nature, more than 90% of BC-related deaths are caused by MBC, which is resistant to conventional anticancer therapy (Mehlen and Puisieux, 2006; Chaffer and Weinberg, 2011). Multidrug resistance (MDR) is a common occurrence in clinical oncology, and one of the most prevalent therapy limits in patients with recurrent malignancies. P-glycoprotein (P-gp) has been linked to the MDR (Li et al., 2010). A drug delivery platform using nanotechnology to treat cancer has been developed that will partially or completely reverse this drug resistance. By inhibiting P-gp, the d-a-tocopheryl polythene glycol 1000 succinate (TPGS) may increase membrane permeability to a drug, improve drug absorption and decrease P-gp-mediated MDR in cancer cells (Constantinou et al., 2008; Ma et al., 2010). Poloxamer 235 was added as a pore-forming agent to improve medication release, in the poly (lactic-*co*-glycolic acid) (PLGA)-TPGS matrix system because the porous PLGA-TPGS/Poloxamer was more likely to be taken up by docetaxel-resistant cells (Tang et al., 2015).

Nanomaterials reduce the occurrence of drug resistance by increasing the uptake of drugs or altering the intracellular distribution of drugs after uptake. Doxorubicin (Dox)-carrying polyester-based hyperbranched dendritic-linear nanoparticles modify subcellular drug distribution by endocytic. Notably, this type of nanoparticle boosted the induction of apoptosis and changed the levels of enzymes and signaling pathways components. Drug resistance can be overcome using polymeric carriers for controlled drug release with significantly greater effectiveness (Zeng et al., 2014). Solid–lipid nanoparticles (SLNs) have attracted great interest as drug carriers because they have biocompatible lipid nuclei and amphiphilic surfactants on the outer surface and benefits from physical stability, prevention of drug degradation, ease of preparation, and low toxicity (Muller et al., 2000). In a study, membrane resistance was circumvented without the aid of chemotherapeutic sensitizers because SLN-loaded Dox could enter the cytoplasm through endocytosis (Chawla and Amiji, 2002). Because SLN-Dox increased the amount of Dox in drug-resistant MCF-7/Adr BC cells, the risk of mortality may be higher (Kang et al., 2010).

Non-specific targeting nanoparticles overcome chemotherapeutic resistance by increasing drug accumulation in cells, although with lower efficiency compared with specific nanoparticles under certain stimuli. For instance, the pH-responsiveness of nanoparticles allowed a model drug to swiftly escape the endosomal system and reach its intended destination. The side carboxyl group on glutamic acid is “on-off” ionized, giving it pH sensitivity. Consequently, the monomethoxy poly (ethylene glycol)-*b*-P (D,L-lactic-*co*-glycolic acid)-*b*-P (L-glutamic acid) (mPEG-PLGA-PGlu) nanoparticle exhibits dual responsiveness compared with free Dox *in vitro*. The pH-dependent and enzyme-sensitive nanomedicine showed increased toxicity and cellular uptake in MCF/Adr cells (Xu et al., 2015). The pH sensitivity of mPEG-PLGA-PGlu nanoparticles can be changed by controlling the length of PGlu fragments. Moreover, owing to dual sensitivity and target accessibility, the nanoparticle-enclosed model drug can quickly escape from the endosomal system. The suppression of autophagy reportedly reduced the tumorigenic capacity of BC stem cells (CSCs) and overcame the radio- or chemoresistance of CSCs (Chaterjee and van Golen, 2011; Kumar et al., 2013; Yue et al., 2013).

Studies have shown that the deletion of genes associated with autophagy, such as LC3, ATG4, and ATG12, or the deletion of autophagy-related genes can lower the CSC subpopulation, inhibit the development of mammospheres, and increase tumor-free survival (Wolf et al., 2013; Yang et al., 2013; Ojha et al., 2015). Nanog, Sox2, and Oct4 were extremely downregulated by autophagy blockade, and autophagy inhibition promoted efficient of chemotherapy against CSCs. Autophagy inhibitors and chemical agents were coupled by Sun *et al.* to synergistically kill both common CSCs and bulk tumor cells. The nanoparticle-based combinatorial delivery system is expected to develop further to enhance the anticancer activity of chemical agents and autophagy inhibitors (Sun et al., 2016).

### 2.2 Nanoparticles enhance antimetastatic effect of chemotherapy

Although various medications are available and useful for treating early stages of BC, not many therapeutic choices are both efficient and pleasant for those who advance to metastatic BC or MBC. Because most conventional chemotherapeutic agents are non-targeted, patients with BC progression or MBC undergo drug changes or use drug combinations, the toxicity of which is difficult to tolerate. The nanocarrier delivery of anticancer drugs protects drugs from degradation, reduces systemic toxicity, and improves biodistribution. Precisely designed nanoparticles with targeting can guide anticancer drugs to the site of action for precision treatment (Zheng et al., 2021). Therefore, the discovery of new precision medications for BC is critical.

**FIGURE 1:**
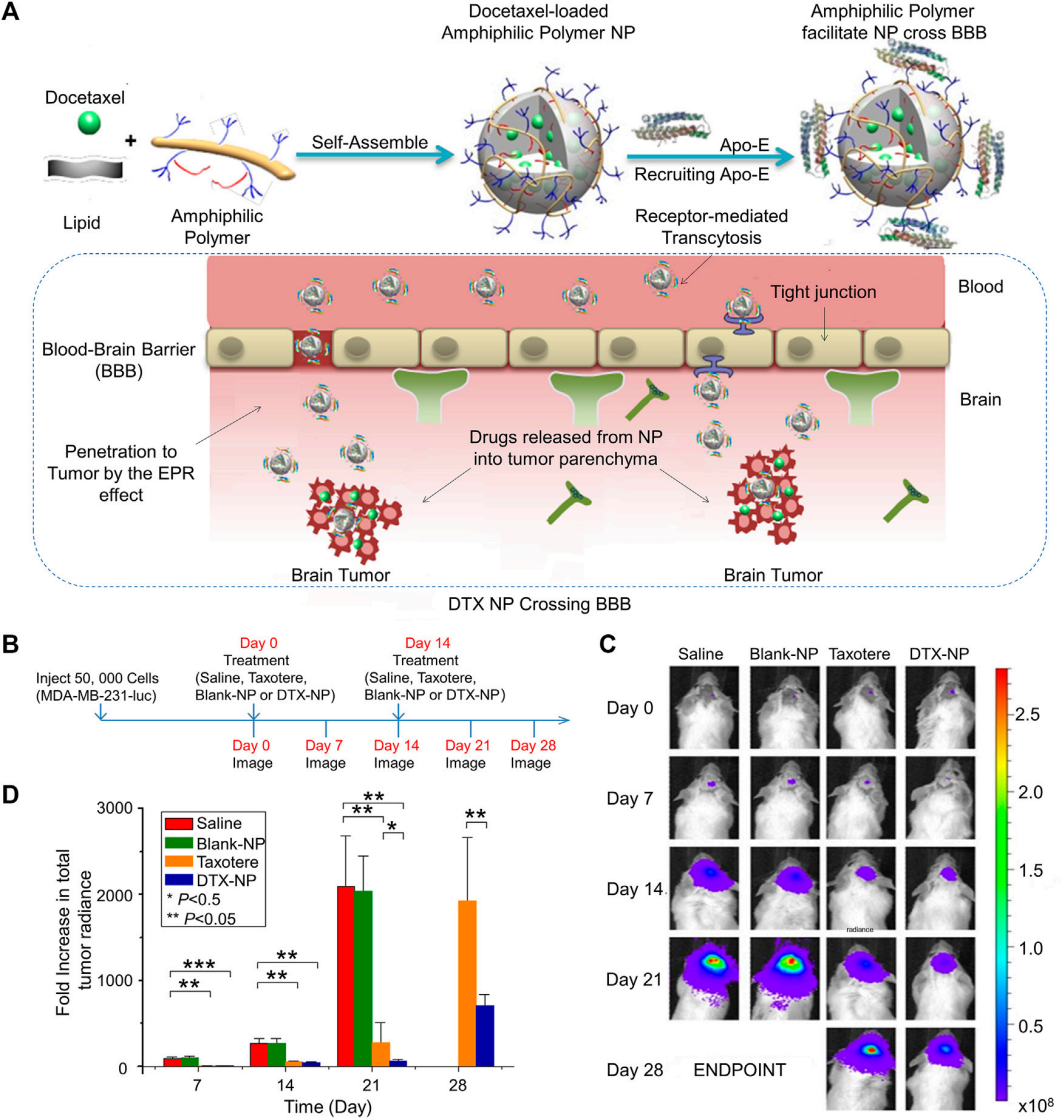
Effective delivery of docetaxel (DTX) by amphiphilic terpolymer nanoparticle (NP) system for the treatment of brain metastasis. **(A)** Schematic illustration of DTX-NP, recruitment of Apo-E, enhanced EPR and breached of the blood-brain barrier. **(B)** Treatment and imaging schedule. **(C)** *In vivo* images of brain tumor bioluminescence. **(D)** An increase of the total tumor radiance. Data are represented as mean ± SD (*n* = 6; *p < 0.05, **p < 0.01, ***p < 0.001). Reproduced with permission (He et al., 2017) Copyright ^©^ 2016 Elsevier B.V.

Brain metastasis is a deadly condition with few available treatments and a very short life expectancy. Systemic chemotherapy although typically ineffective in treating brain metastasis, has a modest effect on peripheral BC metastases. The blood-brain barrier (BBB) is accountable for this resistance. A BBB-penetrating amphiphilic polymer-lipid nanoparticle system was developed to efficiently deliver docetaxel (DTX) for treating of brain metastases in triple negative breast cancer (TNBC). Although DTX and tamoxifen are first-line drugs for treating BC, their different metabolic pathways often cause antagonism The antagonism between the two drugs can be reversed by nanoparticles. Maltodextrin, polysorbate 80 (PS 80), poly (methacrylic acid), and *n*-dodecane form an amphiphilic copolymer that stabilizes ethyl arachidate which formed the nanocarrier (Figure 1A). Treatment with DTX-nanoparticle enhanced DTX circulation dramatically in comparison to Taxotere, and increased the median survival time in animals with brain tumors (He et al., 2017). This is explained by factors such as the improved blood circulation stability of nanoparticles, effective cellular absorption, graded drug metabolism in the tumor, and well-organized drug transport to tumor (Figures 1B–D).

Approximately 70% of patients with MBC have bone metastases, which can cause grave skeletal problems such as pathological fractures, spinal cord compression, or bone discomfort (Coleman, 2006; Buijs and van der Pluijm, 2009). Bone metastasis in BC tend to spread throughout the bone because specific target expression and chemoprevention of the bone microenvironment is lacking (Meads et al., 2008; Croucher et al., 2016). Although initial breast tumors and visceral metastases do not express β3, metastatic bone cancer cells do in preclinical types of BC. Mechanistic investigations have demonstrated that BC-mediated activation of β3 in bone tissue requires transforming growth factor (TGF)-signaling in the Smad2/Smad3 pathway. DTX-containing micellar nanoparticles that are integrin avβ3-targeted (avβ3-phospholipid/polysorbate 80) suppress bone metastasis and tumor-related bone destruction more potently than free-DTX (Ross et al., 2017).

In patients with TNBC, metastasis is the main cause of the death, of which pulmonary metastases accounts for approximately 36.9% (Waks and Winer, 2019), because the lung consists of dense capillaries that facilitates the adhesion of circulating tumor cells (Liang et al., 2020). Tumor proliferation and monitoring of immune evasion are both favored by the immunosuppressive milieu that exists in the lung. Inhaled nanodrugs that deliver anticancer drugs directly to the lungs would be beneficial in inhibiting lung metastases. The treatment of metastatic lesions is also achieved through nanomedicine delivery (Kuzmov and Minko, 2015).

In addition to the important role played by tumor cells in metastasis, their surrounding stromal cells also provide a favorable environment. The development, angiogenesis, and proliferation of tumors are significantly influenced by stromal cells. For instance, fibroblasts subjected to paracrine signals secreted from malignant neoplasms, differentiate into activated cancer-associated fibroblasts (CAFs) (Tarin and Croft, 1969). Most CAFs in the tumor stroma release cytokines that affect both stromal and tumor cells, and they display high levels of smooth muscle actin (α-SMA), which encourages cell division and promotes the malignant phenotype (Cirri and Chiarugi, 2011). Matrix metalloproteinases (MMP), degrade the extracellular matrix (ECM) and promote the migration of tumor and stromal cells into tissues and the bloodstream (Cirri and Chiarugi, 2011; Eckhardt et al., 2012). Stromal cells provide ECM to tissues that have undergone remodeling, constructing a scaffold that supports tumor cell growth.

Cellax was produced through the ester-mediated covalent conjugation of DTX, PEG, and acetylated carboxymethylcellulose. A polymer conjugate that significantly reduces α-SMA levels by 82% or 70% in different BC models, respectively. After therapy, tumor interstitial fluid pressure, tumor matrix, and tumor vascular permeability all rose by more than 30%, 2.5 times, respectively. Approximately, 70-fold increase in tumor perfusion was observed. Cellax treatment reduced lung nodules from 7- to 24-fold, however, native DTX and nab-paclitaxel (PTX) therapies were ineffective. A much better antimetastatic outcome was observed with the antistromal action of Cellax treatment. These results support the more efficient targeting of tumor stroma by Cellax (Murakami et al., 2013).

## 3 Nanoparticle-based gene therapy

Compounds, such as monoclonal antibodies, proteins, peptides, nucleic acid aptamers, polysaccharides, and small molecules, can serve as targeting moieties (Chen et al., 2013; Noble et al., 2014). Monoclonal antibodies and small-molecule inhibitors are examples of conventional medicines. These compounds can only impact a small subset of proteins and processes, or “druggable” targets. Only extracellular or proteins that have been liberated from the cell membrane are targeted by monoclonal antibodies. siRNAs are frequently used to silence genes because RNA interference is a dependable and established technique. The inhibition of oncoprotein expression at the mRNA level inhibits the synthesis of active proteins, making RNA interference more effective than monoclonal antibodies and small-molecule inhibitors, which only restrict the activity without preventing the production of new active oncoproteins.

Since 1998, siRNA has been used for down regulating target genes expression. All genes responsible for malignancy hallmarks, such as angiogenesis, invasion, and metastasis, can be targeted for silencing. Because of the anionic charge and the large molecular weight of its phosphodiester backbone, it is challenging for siRNA to traverse negatively charged cell membranes (Wang et al., 2010). Moreover, siRNA has low cellular absorption and a considerably short blood half-life in the blood, delivering siRNA to tumors *in vivo* has proven challenging (Choi et al., 2007; Wang et al., 2010). Additionally, unmodified siRNAs can activate the innate immune system. To overcome these difficulties, siRNA needs to be further shielded when used in a therapeutic environment.

The delivery of siRNA using nanoparticles is the most promising method available for treating cancer. Patisiran (Alnylam), the first siRNA therapeutic approved by the US Food and Drug Administration (FDA), was used to manage heritable transthyretin-mediated amyloidosis (Adams et al., 2018; Ledford, 2018). This positive outcome supports the potential use of siRNA technology in therapeutic applications. Potentially, systemic clearance may be slowed by siRNA-loaded nanoparticles with a size range of 30–200 nm. Endosomal escape is a significant cellular obstacle for siRNA delivery (Dominska and Dykxhoorn, 2010). Nanoparticles must be able to damage endosomal membranes to release from the endosome and enter the cytosol, where they may function. To overcome these challenges, various siRNA modification techniques have been developed; however, these modifications may reduce the effectiveness of siRNA-mediated silencing (Bramsen et al., 2009; Pascut et al., 2015).

Different elements of cancer can be simultaneously targeted through multi-siRNA delivery using nanoparticles. This need can be met by simultaneously knocking down several genes by delivering different siRNAs on different nanoparticles to the same tumor.

### 3.1 Nanoparticles overcome resistance to gene therapy

Because the HER2 oncogene and associated genetic components amplifying in the amplicon on the chromosome, the clinical subtype known as HER2+ BC displays HER2 overexpression on the tumor cell surface (Nassar et al., 2014). Between 15% and 25% of invasive BCs fall under this clinical subgroup (Prat et al., 2014). Clinically, between 20% and 50% more cases of brain metastasis occur in patients with HER2 or triple-negative form of BC (Kennecke et al., 2010; Aversa et al., 2014).

For systemic administration of siHER2, Ngamcherdtrakul *et al.* created a delivery system based on mesoporous silica nanoparticles (MSNP) with trastuzumab as the target drug. The nano-construction comprised cross-linked polyethylenimine (PEI) and PEG surface modification (50 nm) on top of an MSNP core. Although PEI boosted the endosomal escape of siRNA, PEG enhanced overall blood compatibility, provided steric effects to avoid aggregation, and shielded siRNA from enzyme-mediated degradation. Trastuzumab was further conjugated to improve selectivity and homing abilities (Ngamcherdtrakul et al., 2015). Based on these findings, mesoporous silica nanoparticles were developed to concurrently distribute trastuzumab, DTX, and siHER2. When combined with targeted ultrasound aided by microbubbles, trastuzumab–siHER2–NP(DTX) effectively caused a therapeutic effect in mouse brain breast tumors (Ngamcherdtrakul et al., 2022). The treatment of brain metastases from HER2+ BC may be improved by use of nanomaterials. This adaptable nanoparticle platform may combine the administration of multiple treatment modalities, ensuring that they reach the target cells simultaneously to provide synergistic effects.

### 3.2 Nanoparticles enhance the antimetastatic effect of gene therapy

**FIGURE 2:**
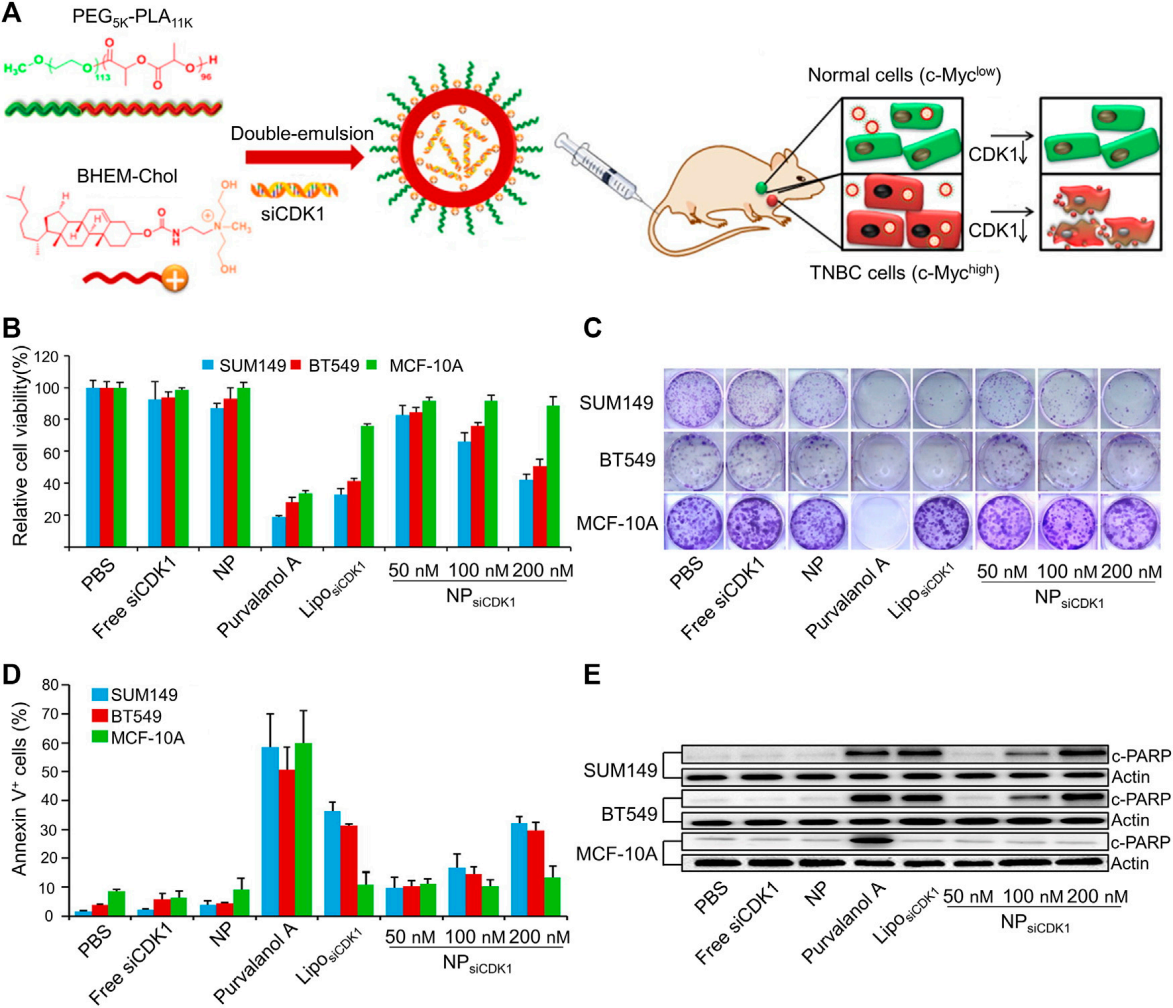
Cationic lipid-assisted PEG-PLA nanoparticle system delivers siCDK1 for the treatment of triple-negative breast cancer (TNBC) with over-expressed of c-Myc. **(A)** Schematic illustration of nanoparticles. **(B)** Cell viability and **(C)** colony formation of cells. **(D)** Cell apoptosis and **(E)** c-PARP expression change. Reproduced with permission (Liu et al., 2014) Copyright ^©^ 2014, Elsevier B.V.

siRNA uses a precise mechanism to achieve protein knockdown. Multiple siRNA nanoparticle delivery systems have been created to limit BC metastasis. In a study, the inhibition of the NF-κB pathway, inhibited the expression of the enzyme matrix metalloproteinase-9, which degrades the ECM and basement membrane and promotes cancer cell extravasation (Deryugina and Quigley, 2006). In 4T1 cells, migration and invasion were prevented by blocking the NF-kB pathway (Yu et al., 2016). Yu *et al.* used a micelle based on a polymer to deliver siRNA against the NF-kB component p65 (sip65) poly (ethylene glycol)-block-poly (aminolated glycidyl methacrylate) (aminolated glycidyl methacrylate) poly (2 (diisopropylamino) ethyl methacrylate) block polymer (PEG-*b*-PAGA-*b*-PDPA) triblock copolymers and found that the sip65 delivered by this micelle prevented the orthotopically implanted 4T1 tumors from metastasizing to the lung (Yu et al., 2016). In TNBC, the oncoprotein Myc is frequently overexpressed. To deliver cyclin-dependent kinase 1 (CDK1) siRNA to TNBC cells with elevated Myc expression, Liu *et al.* used a cationic lipid-based PEG-polylactic acid (PEG-PLA) nanoparticle (Figure 2A). *In vitro*, decreased Myc expression has been linked to impaired colony formation, decreased cell viability, and increased apoptosis (Figures 2B–E) (Liu et al., 2014).

Previous research has shown that TGF-mediated epithelial-mesenchymal transition (EMT) depends on β3 integrin (Parvani et al., 2013). The epithelial markers cytokeratin (CK)19 and E-cadherin are upregulated, and N-cadherin and PAI1 are downregulated when β3 integrin is silenced *in vitro* (Parvani et al., 2015). Increased circulation time and tumor cell absorption were achieved by adding PEG, which was coupled to an RGD peptide that is recognized by β3 integrin, to the lipid ECO-based nanoparticles that carry β3 integrin siRNA (Iyer et al., 2013). RGD-ECO/siβ3 lipid nanoparticles significantly reduced the burden of the main tumor, primary tumor recurrence, and metastatic tumor in nude mice engrafted with TNBC (Parvani et al., 2015).

BC cells that have spread to the brain release C-C chemokine ligand 2 (CCL2), which attracts myeloid cells and aids in the formation of metastatic tumors [66]. Nanoparticle-siRNAs, reduced the amount of tumor in mouse brains by knocking down long non-coding RNA associated with BCBM (Lnc-BM) *in vivo*. Lnc-BM caused the release of CCL2 by activating a downstream signaling pathway, and Lnc-BM/JAK2/STAT3 was involved in generating positive feedback [67].

## 4 Nanoparticles enhance immunotherapy

### 4.1 Nanoparticles enhance innate immunity

Nanotechnology has the potential to be beneficial in reversing immunosuppression within the TME (Mao et al., 2020). Immunosuppression frequently renders the anticancer immune response ineffective, allowing some cancers to evade immune monitoring and spread. The most studied immune evasion mechanisms in BC include the expression of inhibitory co-stimulatory molecules, tumor-associated macrophages (TAM) in the microenvironment, maturation of dendritic cells (DC), killing activity of natural killer (NK) cells, and the presence of inhibitory factors.

#### 4.1.1 Macrophages

Macrophages are one of the most prevalent immune cells in BC. Increased macrophage density is associated with many clinical traits, such as invasiveness, metastasis, immunosuppression, neovascularization, and a subpar response to treatment. TAMs are mostly proinflammatory macrophages that accelerate tumor growth by generating proangiogenic and inflammatory cytokines during the initial stage of tumor development (De Palma et al., 2017; Salmaninejad et al., 2019). Targeting and reprogramming tumor macrophages can enhance immunotherapy (Franklin et al., 2014). In cancer, macrophages exhibit a continuum of activation states with two extremes. Traditionally activated M1-like macrophages (M1 cells) modify host defense against infections and activate antitumor immunity. However, a protumorigenic response can be mediated by activated M2-like macrophages (M2 cells), which do so by boosting angiogenesis and reducing the cytotoxic immune response. As a result, a novel and potential treatment approach involves changing the tumor immune milieu by altering TAM polarization.

**FIGURE 3:**
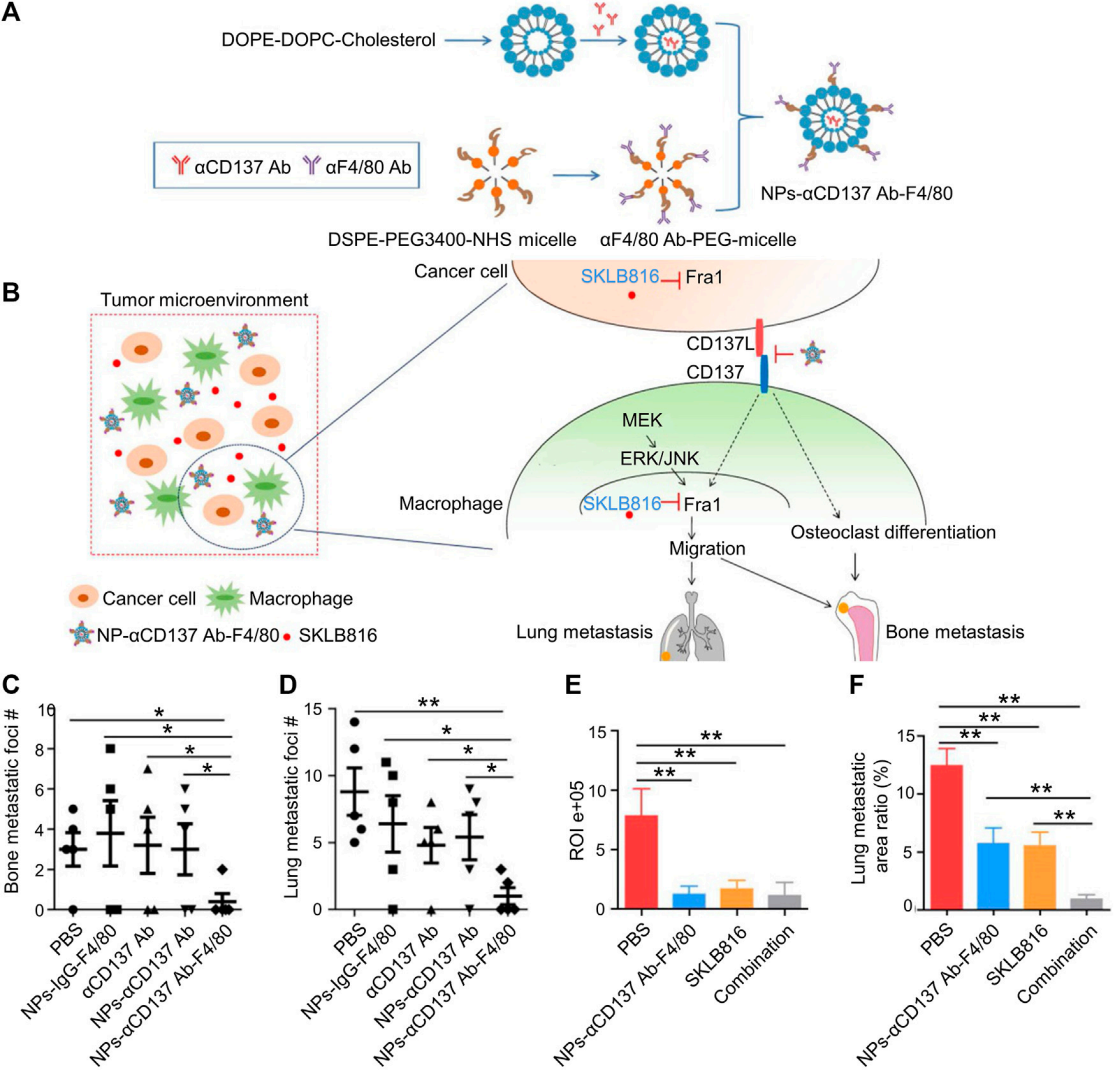
Dual-target therapy against CD137 and Fra1 for BC. **(A)** Schematic illustration for the preparation of NPs-αCD137 Ab-F4/80. **(B)** Schematic summary of treatment model: NPs-CD137 Ab-F4/80 suppress osteoclasts and lessen osteoclast migration by blocking CD137 signaling in macrophages. **(C–D)** Statistical results of the number of metastases in lungs and bones in NPs-αCD137 Ab-F4/80 treatment (*n* = 5 mice). **(E)** Statistical results of normalized BLI signals (*n* = 4 mice). **(F)** Lung metastasis area ratio results (*n* = 4 mice), Data are represented as mean ± SD (*p < 0.05, **p < 0.01). Reproduced with permission (Jiang et al., 2019) Copyright ^©^ 2019, Ivyspring.

CD137, mostly expressed in activated leukocytes, belongs to the superfamily of the tumor necrosis factor (TNF) receptor. CD137 promotes monocyte and macrophage migration to the TME by increasing Fra1 expression. An F4/80-targeted liposomal nanoparticle was created that contained the anti-CD137 blocking antibody (Figures 3A,B). BC metastases to the bones and lungs were considerably reduced in mice when F4/80 + monocytes/macrophages were depleted (Figures 3C–F). Targeting F4/80 + macrophages and suppressing their CD137 signaling may successfully prevent the occurrence of tartrate-resistant acid phosphatase positive osteoclasts in the bone metastatic lesions of BC (Jiang et al., 2019).

Nanosized therapies are unable to penetrate efficiently due to a robust ECM and significant interstitial fluid pressure. An acid-triggered size-changeable nanoparticle (aptamer/acid sensitive linker crosslinked dendrigraft poly-L-lysine (DGL)/zoledronic acid, or Apt@(DGL-ZA)n nanoparticles) with effective tumor dispersion, extravasation, and penetration was developed to solve this problem. DGL was crosslinked using a mild-acid responsive linker [1,6-bis(4-formylbenzoyloxy) hexane], which resembles natural aberrant proteins to promote tumor autophagy. Charge attraction loaded zoledronic acid (ZA), a macrophage conditioning agent onto DGLs. For a tumor-homing effect to (DGL-ZA)n nanoparticles, a tenascin-C targeting aptamer (GBI-10) was modified. The results showed excellent *in vivo* anticancer effectiveness, improved tumor autophagy, and effective macrophage control (Guo et al., 2019).

To provide siRNAs to tumor M2-TAMs and BC cells that target vascular endothelial growth factor (VEGF) and placental growth factor (PIGF), Song *et al.* developed a polymeric nanoparticle based on PEG- and mannose-doubly modified trimethyl chitosan and citraconic anhydride grafted poly (allylamine hydrochloride) (Song et al., 2018). In tumors, low pH conditions trigger PEG lysis, exposing mannose and cations to particles. Nanoparticles promote the transport of macrophages and cancer cells in tumors while repolarizing M2 macrophages to M1 macrophages.

The biodegradable poly (ε-caprolactone)-*b*-poly (2-aminoethylethylene phosphate) (PCL-*b*-PPEEA), PEG-*b*-poly (ε-caprolactone) (PEG-*b*-PCL), and PCL homopolymer used in RNAi nanoparticles can prevent BC from spreading. CCL-18 is a possible therapeutic target, which is released by TAMs and triggers cancer cell EMT, boosts BC metastasis, and lowers patient survival. The best nanoparticle platform can effectively reduce the expression of CCL-18 in macrophages, thereby reducing the migration of BC cells (Liang et al., 2018).

#### 4.1.2 Dendritic cells

Vaccines achieve the induction, regulation and maintenance of T cell immunity through DCs (Hamdy et al., 2011). Polymeric nanoparticles can improve the efficiency of tumor vaccine delivery systems. Cell membrane cancer immune nano-vaccines are effective in inducing tumor-specific immunity. In addition, PLGA NPs are used together for cancer prevention and treatment (Jiang et al., 2020). In addition to the breakthroughs made outside of vaccines, the advantages of nano-systems for drug delivery have emerged in recent years. Endoglin-binding peptide (EBP), Dox, the immunomodulator polyinosinic (polycytidylic acid), the tumor and vascular target ligand, and the iron oxide core were chosen as a reliable shape-defining template for conjugations to minimize size (PolyI:C). Both TNBC cells and vascular epithelia are targeted by EBP. The nanoparticle promoted tumor apoptosis through various methods, including direct tumor cell death, DC mediated innate immune responses started by DCs, and T cell-mediated adaptive immune responses. The nanoparticle significantly improved survival in an aggressive and drug-resistant metastatic TNBC animal model and significantly reduced tumor growth and metastasis (Mu et al., 2021).

“Ion trapping”, is a phenomenon associated with the extracellular acidic pH that creates a physiological drug barrier, thereby impacting on tumor chemosensitivity. Because Dox is a weak base, it experiences ion trapping in the acidic tumor environment, which decreases its cellular uptake and inhibits its permeation through lipid cell membranes (Raghunand et al., 1999; Mahoney et al., 2003; Yoneda et al., 2015). Hyaluronic acid (HA) and Dox were linked to form the polymeric prodrug HA-Dox by an acid-cleavable hydrazone bond linkage. Then, HA-Dox/PHIS/R848 nanoparticles were deposited onto nanocores. The HA-Dox/PHIS/R848 nanoparticle system, which targets both immune cells and cancer cells, was developed to integrate immunotherapy with chemotherapy to treat BC. In mice, HA-Dox/PHIS/R848 nanoparticles demonstrated exceptional tumor-targeting abilities by reducing tumor immunity and eradicating tumor cells (Liu Y. et al., 2018).

#### 4.1.3 Natural killer cells

Given that they are less likely than T cell-based treatments to result in unfavorable events, such as cytokine storms or graft-versus-host disease, NK cells were identified as a potential treatment for cancer. Although cytokine therapy and genetic engineering to activate NK cells have been researched extensively, the approaches are inefficient, expensive, and labor-intensive. Clinically, there is a strong correlation between the beginning of malignancy and limited NK cytotoxicity (Imai et al., 2000). By contrast, a significant number of tumor-infiltrating NK cells is a reliable indicator of good prognosis for in patients with cancer (Ishigami et al., 2000). The efficiency of NK cell-mediated immunity treatments hinges on the balancing act between inhibitory and stimulatory signals sent by receptors and ligands to which they bind. Advances in nanotechnology have facilitated the development of nanoparticle technology acting on NK cells. Previously, cytokines or other stimulating chemicals were encapsulated in nanoparticles and then delivered to NK cells to activate them (Nakamura et al., 2018; Phung et al., 2020). Studies have demonstrated that cationic polymer-based nanoparticles promote the generation of proinflammatory cytokines and elicit a strong humoral response (Wegmann et al., 2012; Yim et al., 2014; Mulens-Arias et al., 2015; Li et al., 2018).

Cationic nanoparticles (cNPs) were generated by employing PDA chemistry to immobilize PEI on the surface of magnetic nanoparticles. *In vitro*, the cytotoxic activity of cNP-treated primary NK and NK-92MI cells against TNBC cells was more than two-fold that of control NK cells. Molecular analysis revealed that, cNPs changed the way CCR4 and CXCR4 chemokine receptors were expressed on NK cells. *In vivo* TNBC animal models showed significant tumor growth inhibition by cNP-treated NK cells. The approach offers a promising framework for NK cell-based *ex vivo* cancer treatment (Kim et al., 2020). Exposed after the acid challenge because of the hydrophilicity and low molecular weight of HEMA (Moreira et al., 2018; Hu et al., 2021).

#### 4.1.4 Nanoparticle promote adaptive immunity

The most widely used programmed cell death protein 1 and programmed cell death-ligand 1 (PD-1/PD-L1) blocking medication only has a 20% response rate in PD-L1 positive TNBC (Adams et al., 2019). There is an urgent need to develop strategies that complement PD-1/PD-L1 blockade therapy to boost the effectiveness of immunotherapy and reverse the immunosuppressive TME.

The immunological checkpoint receptor PD1, which has received considerable attention, is primarily expressed on the surface of T lymphocytes and is overexpressed on the surfaces of depleted T cells. PD-L1 is expressed primarily on the surface of solid tumors cells. The association between PD1 and PD-L1 inhibits T cell growth and activation by inhibiting kinase signaling pathways (Nixon and Li, 2017). T cells are the most effective antitumor effector cells. The number of intratumoral CD8^+^ T cells is critical after treatment because it correlates with improved immunotherapy and chemotherapy responses and prolonged patient life.

However, the off-target effects of the antibodies can cause immune-related adverse outcomes in the skin and liver and gastrointestinal, endocrine, and respiratory systems (Topalian et al., 2012; Topalian et al., 2019). In addition to the use of antibodies, effective delivery of siRNA can inhibit PD-L1, thereby inhibiting the interaction between PD-L1 and PD1. Studies have revealed that siPD1/PD-L1 increases the antitumor effects of chemotherapeutic drugs (Liu B. et al., 2018).

siPD-L1 and an indoleamine 2,3-dioxygenase inhibitor were delivered through a nano-delivery that contained with a BC homing and penetrating peptide as a dual immune checkpoint blocker. The siRNA was endocytosed by tumor cells but, escaped the endosomal vesicles because the vector could home to BC cells. The medication then prevented the metabolism of tryptophan in these cells. BC cells died as a result of the locally produced siPD-L1 and 1-methyl-DL-tryptophan, which encouraged the survival and activation of cytotoxic T lymphocytes (CTLs) (Li et al., 2019).

In a study, tumor infiltrating lymphocytes (TILs) and MCF-7 cells were treated with siRNAs against PD-L1 and PD1 using lipid-coated calcium phosphate nanoparticles. siRNA significantly increased TIL cytotoxicity in cancer cells by downregulating PD1 and PD-L1. Combining PD1 and PD-L1 knockdown was more effective in boosting the TILs ability to kill cancer cells than either one alone. Combined PD1 and PD-L1 silencing increased the secretion of proinflammatory cytokines, such as interferon-γ (IFN-γ) and tumor necrosis factor alpha (TNF-α) (Prima et al., 2017).

The use of nanomaterials not only improves the efficiency of siRNA delivery, but also enhances the therapeutic efficacy of antibodies. For temporary chemo-immunotherapy against lung metastasis, Zhao *et al* created “walking dead” TNBC cells by genetically modifying cell corpses with PD-1 and Dox-loaded liposomes. Disulfide linkages were used to couple PD-1 to the cell, allowing for reduction-triggered release onto activated T lymphocytes. Maleimide-thiol coupling, which secures the Dox-loaded liposome to the cell, enabled the continuous release of Dox. The walking dead TNBC cells increase mouse survival by generating a local drug delivery depot, extending drug retention duration, and greatly suppressing lung metastasis, thereby promoting lung metastasis-targeted drug delivery (Zhao et al., 2022).

**FIGURE 4:**
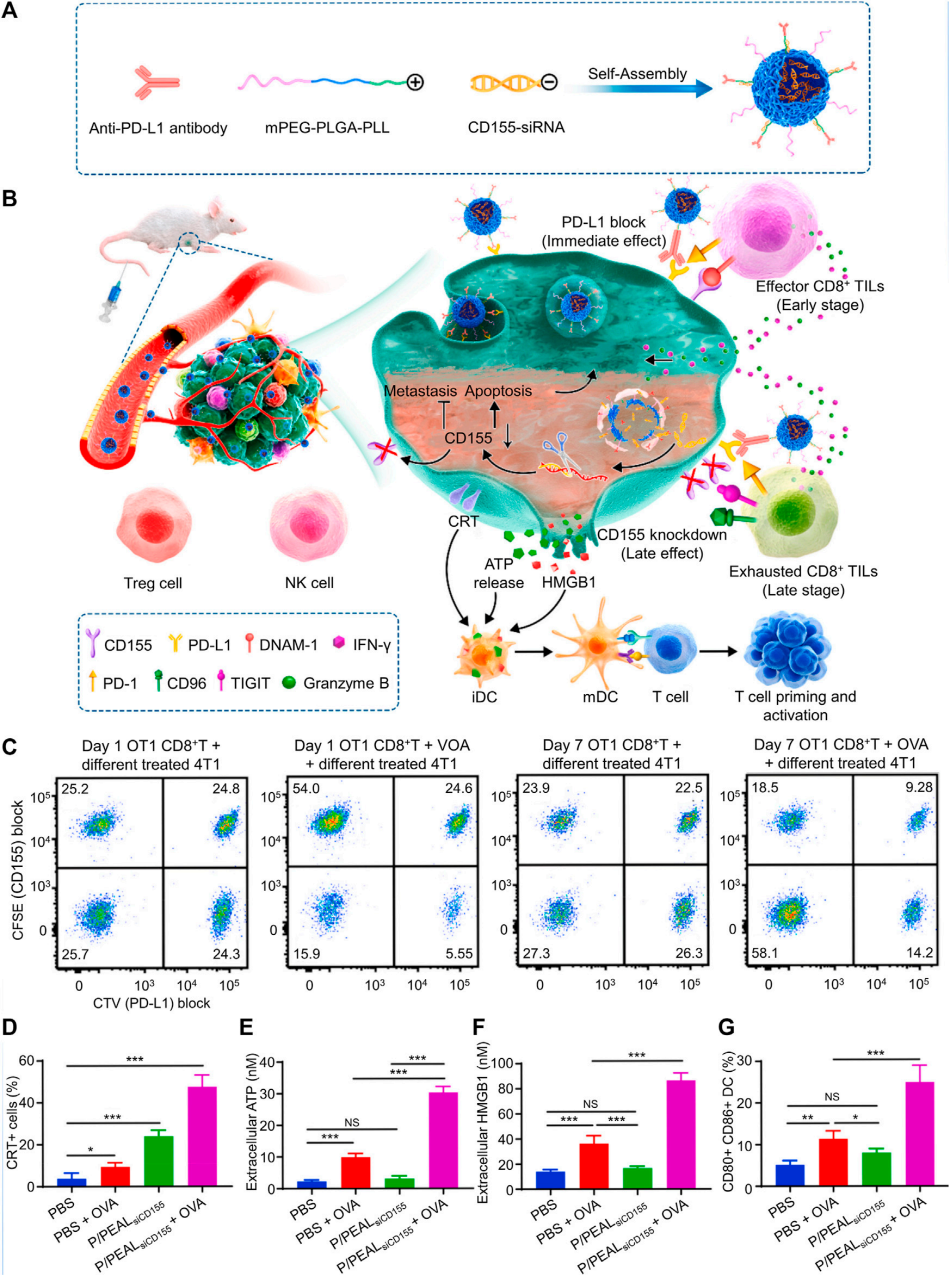
Immune checkpoint therapy was made easier by P/PEAL_siCD155_, which asynchronously inhibited PD-L1 and caused immunogenic cell death (ICD) in a spatiotemporal way. **(A)** Schematic illustration of P/PEAL_siCD155_. **(B)** Schematic representation of the treatment of P/PEAL_siCD155_-mediated combined immunotherapy in tumor model. **(C)** Examining the CD155 and PD-L1 intensity to determine the percentage of 4T1 cells that were subjected to various treatments. **(D–G)** ICD in 4T1 cells including CRT **(D)**, ATP **(E)**, and HMGB1 **(F)** is triggered by P/PEAL_siCD155_. **(G)** DC maturation *in vitro*. Data are represented as mean ± SD (*n* = 3; *p < 0.05, **p < 0.01, ***p < 0.001). Reproduced with permission (Chen et al., 2021) Copyright ^©^ 2023, Elsevier.

Using positively charged mPEG-PLGA-PLL (PEAL) as the backbone, P/PEAL_siCD155_ polymeric nanoparticles were created for PD-L1 and CD155 asynchronous blockade (Figure 4A). In CD8^+^ TILs, PD-1 and DNAM-1 were upregulated early, whereas CD96 and TIGIT were upregulated later (Figure 4B). In the 4T1 orthotopic model, P/PEAL_siCD155_ NPs may modify the CD155/DNAM-1, CD155/TIGIT, CD96, and PD-L1/PD-1 axis to prevent the spread of TNBC and block CD155-mediated immune surveillance in the early stages (Figures 4C–G) (Chen et al., 2021).

Immune checkpoints control CTL activity negatively. CTLs are the primary effector cells that mediate immune antitumor responses and identify tumor antigens. The interaction between co-stimulatory pathways and checkpoints controls CTL activity. After the tumor antigen is presented, the B7 (CD80/CD86) receptor on the surface of APCs is bound by the CTL-associated CD28 receptor, thereby activating CTL. Then, the cytotoxic T lymphocyte associated antigen 4 (CTLA4) is transferred to the CTL membrane, where it competes with CD28 for B7 and attaches to it with a greater affinity, blocking the previously active pathways (Qureshi et al., 2011). In patients with cancer, CTLA4 overexpression is a key immune evasion mechanism. Initially, CTLA4 was thought to be a T cell-associated molecule. Nevertheless, data suggests that CTLA4 is expressed in numerous non-lymphoid cell types (Mao et al., 2010). Additionally, normal breast tissues do not express CTLA4, although ∼50% BCs do (Kassardjian et al., 2018). CTLA4 inhibits the development of T cell effector function by promoting transendocytosis and ligand degradation (Krummel and Allison, 1995). CTLA4 induces T cell tolerance by inhibiting T cell expansion and IL-2 release (Greenwald et al., 2001; Qureshi et al., 2011). CTLA4 balances T cell receptors (TCRs)/CD3-mediated phosphorylation and prevents TCR signal transmission through the immunoreceptor tyrosine-based inhibitory motif (ITIM) (Rudd et al., 2009). Thus, CTLA4 participates in a large population of T lymphocytes and acts as an essential immune checkpoint, ultimately inhibiting T cell effector functions and suppressing antitumor immune responses.

A nanoparticle comprising three FDA-approved substances—PLGA, indocyanine green (ICG), and imiquimod (R837) was developed to increase the immunotherapy efficacy of anti-CTLA4 checkpoint-blockade. Although R837 is a powerful TLR7 agonist that triggers immunological responses, ICG serves as the near-infrared dye to enable photothermal therapy (Kasturi et al., 2011; Le Mercier et al., 2013). The released tumor-associated antigens and R837-loaded nanoparticle adjuvant would demonstrate vaccine-like functions upon near-infrared-induced photothermal ablation of primary tumors injected with PLGA–ICG–R837, resulting in potent immune reactions, which with the aid of the anti-CTLA4 checkpoint-blockade would inhibit the immune suppressive Tregs could target distant tumor cells present in the mouse. The immune memory protected mice from cancer recurrence, as demonstrated by the ability of anti-CTLA4 therapy in conjunction with PLGA–ICG–R837–based photothermal treatment to protect treated mice from tumor cells rechallenged 40 days after first tumor ablation. This therapy.

The immunosuppressive molecules that seem to restrict TILs *in vivo* include T cell immunoglobulin and mucin domain-containing protein 3, lymphocyte activation gene 3 protein, PD1/PD-L1, and CTLA4 (Watanabe et al., 2003; Curran et al., 2011; Woo et al., 2012). Blocking these negative regulators on T cells using nanomaterials enhances the antitumor T cell response and thus improves the outcome of immunotherapy for patients with cancer.

### 4.2 Cytokine therapy

In the TME, VEGF-a and VEGF-c play crucial roles in angiogenesis and lymphangiogenesis, respectively (Kadowaki et al., 2005). Chemokines, in particularly CCL2, produced by both cancerous and stromal cells, control the recruitment of TAMs to solid tumors (Saji et al., 2001; Ben-Baruch, 2003). The CCL2 system and its main receptor CCR2 were reported to encourage tumor cell survival and motility (Fang et al., 2012), metastasis (Qian et al., 2011), and angiogenesis (Saji et al., 2001). Immunogenic chemotherapy mediated the anticancer immune response, and CCL2 mediated monocyte or myeloid cell recruitment (Ma et al., 2014). As a compensatory mechanism, CCL2 has been shown to counteract the antitumor effects of vascular-targeted therapies, such as VEGF inhibitors (Bergers and Hanahan, 2008).

Numerous tumor cell types, including MCF-7, have been found to overexpress somatostatin receptors (SSTRs), especially SSTR subtype 2 (SSTR2) (Bruns et al., 1994; Kahan et al., 1999; Kumar et al., 2005). Vapreotide (RC-161) is an endogenous somatostatin octapeptide analog with a strong affinity for SSTR2 (Coy and Taylor, 1996). The PLPC/siRNA nanoparticle was composed of a chondroitin sulfate core with a negative charge that was condensed by protamine and coated with a cationic lipid shell that contains the hydrophobic PTX. PEG phospholipid (DSPE-PEG) and/or vapreotide were then added to the surface of the lipid-shell nanoparticle. The targeted and non-targeted PEG-PLPC/siRNA nanoparticles had similar physicochemical properties. However, the VAP-PLPC/siRNA nanoparticles significantly slowed tumor development (Feng et al., 2014).

Tumor angiogenesis is essential for tumor growth and metastasis. Antiangiogenic therapy alone did not achieve powerful and long-lasting therapeutic effects. To treat BC metastases by combining antiangiogenesis and immunological activation, RGD-PEG-*b*-PGA-g-(TETA-DTC-PHis) (RPTDH), a copper chelating coil-comb block copolymer, was developed and used to make nanoparticles for loading R848, a TLR7 and TLR8 agonist. RPTDH/R848 nanoparticles demonstrated excellent targeting ability against primary BC and lung metastases. Zhou *et al* reported a dramatic reduction of tumor growth and metastasis by inducing antiangiogenesis in response to copper deficiency and immune activation in response to R848 (Zhou et al., 2019).

PTX inhibits primary tumor growth, but there is accumulating evidence that it may encourage metastasis by increasing proinflammatory cell-free nucleic acids levels that damaged cells discharge into the TEM (Wu et al., 2019). Polymeric nanoparticles that administer chemotherapeutics while scavenging proinflammatory factors are being developed to limit chemotherapy-induced BC spread. Based on charge–charge interactions, cationic polyamidoamine (PAMAM) dendrimers modified with drug-binding dodecyl groups and surface groups of diethylethanolamine (PAMAM-G_3_-C_125_-DEEA_20_) can adsorb cell-free nucleic acids, which downregulate TLR expression, and thus, reduce inflammatory cytokine secretion. Cancer progression was inhibited at both the primary and metastatic locations by encapsulating a chemotherapy in a cationic nanocarrier (Li et al., 2022).

## 5 Summary, future challenges, and opportunities

BC is a common cancer with high incidence that endangers women health. Nanoparticle-mediated therapies can be an effective substitute for conventional treatment methods, including surgery, radiation therapy, chemotherapy, hormone therapy, and immunotherapy. Nanomaterials enable prolonged drug activity with precise and controlled drug targeting to overcome the limitations of BC. Targeted and non-targeted nanocarrier-mediated transport of anticancer drugs has yielded promising results in the treating BC (Jiang et al., 2022).

Nanoparticle-based drug delivery is a promising new strategy. Targeted drug or gene delivery with BC treating nanomedicine is rapidly developing and promises to overcome the drawbacks of traditional treatments. Nanoparticles have the potential to revolutionize BC gene therapy because they can effectively transport a drug or gene by increasing circulation time, increasing bioavailability, lowering immune detection, and increasing delivering accuracy. Systemic administration of siRNAs is considered more relevant and practicable than local treatment to target a wider range of malignancies, including advanced cancer or metastasis.

Drug resistance and metastasis are a serious challenge in BC subtypes. All types of BC have responded to combination therapy, but cancer cells are exceptionally skilled in altering signaling pathways and thwarting pharmaceuticals from reaching their targets. Nanodelivery systems reverse drug resistance and metastasis by altering the mode of action of drugs through their own biological characteristics. Furthermore, conventional BC combination therapy regimens are based on sequential and independent dosage. The time window for drug cohabitation over therapeutic levels in the plasma and tissues is often small, and pharmacokinetics may increase resistance in cancer cells while producing toxicity in healthy tissues. The limitations of chemotherapy will be circumvented by deploying rationally designed combination therapeutic vehicles that preferentially target cancer cells. Advanced drug delivery technologies will be able to simultaneously deliver the drugs in the combination regimen to the body, synchronizing pharmacokinetics. Not all drugs are suitable for combination from a pharmacological point of view. A system that is pH or redox sensitive is unstable in the internal environment. As a consequence, combining a drug with nanomaterials may not always be effective. Therefore, the drug’s mode of action, pharmacokinetics, and other factors must be evaluated.

The main application of nanotechnology includes fabrication of nanoparticles with various components that may be useful in the fight against cancer. Delivery of nanoparticles improves cancer therapy effectiveness while reducing toxicity to normal cells. Another possible strategy for treating MBC is nanoparticle-mediated immunotherapy, which has shown good outcomes in preclinical investigations. The involvement of scientists from the disciplines of physics, engineering, and chemistry has helped develop nanoparticle-based immunotherapy for BC. Fortunately, the BC treatment industry recognizes the promise of nanoparticles, and investment is increasing rapidly. However, many uncertainties prevail that must be studied and many obstacles need to be overcome. Despite the difficulties at this stage, the development of effective clinical applications of nanomedicines is not impossible, but will require interdisciplinary collaboration.
